# Place & Person involved in delivery: Factors leading to delay in diagnosis of Anorectal Malformation in Newborns

**DOI:** 10.12669/pjms.38.1.4156

**Published:** 2022

**Authors:** Shazia Perveen, Sajid Ali, Abdul Jabbar, Batool Fatima

**Affiliations:** 1 Dr. Shazia Perveen, FCPS. Department of Pediatric Surgery, National Institute of Child Health, Jinnah Sindh Medical University, Karachi, Pakistan; 2 Dr. Sajid Ali, FCPS. Department of Pediatric Surgery, National Institute of Child Health, Jinnah Sindh Medical University, Karachi, Pakistan; 3 Dr. Abdul Jabbar, Resident, Department of Pediatric Surgery, National Institute of Child Health, Jinnah Sindh Medical University, Karachi, Pakistan; 4 Dr. Batool Fatima, Resident, Department of Pediatric Surgery, National Institute of Child Health, Jinnah Sindh Medical University, Karachi, Pakistan

**Keywords:** Anorectal Malformations, Delayed diagnosis of Anorectal malformations, Hospital delivery, Home delivery, Person detecting Anorectal Malformations

## Abstract

**Objective:**

To determine the frequency of place of delivery and person detecting the anomaly among newborn babies presenting with delayed diagnosis of anorectal malformation (ARM).

**Methods:**

This is a Descriptive Cross-Sectional Study, conducted at Department of Paediatric Surgery, National Institute of Child Health (NICH) Karachi, from February 19, 2019 to August 18, 2019. All patients with ARM who were diagnosed beyond two hours of life (Delayed diagnosis) were included in the study. Chi square test was applied for comparison of categorical variables.

**Results:**

Total 110 patients were enrolled in this study. Nineteen (17.3%) patients were delivered at home, while 91 (82.7%) were delivered at the hospital. The first person detecting the anomaly was grandmother (n=25) or a non-medical person in 58 patients (52.7%), 52 were diagnosed by a medical personal either primarily in 31 cases (28.2%) or secondarily by a neonatologist in 21 cases (19.1%).

**Conclusion:**

It is concluded that Non-medical person detected ARM mainly despite the babies being delivered mostly at the hospital, indicating the need for meticulous neonatal examination.

## INTRODUCTION

An Anorectal malformation (ARM) is absence of normal anal opening due to maldevelopment of anorectal canal with or without associated fistula to genitourinary tract or to exterior. It comprises a wide spectrum of conditions that affects the anorectal development in either gender. Estimated incidence of anorectal malformations is 1 per 4000 to 5000 live births.^1^ The population-based data of ARM from Asia is not available. In a study conducted at Lahore Pakistan, managing 100 newborns with ARM over eleven months’ period, points towards significant disease burden in Pakistan.^2^

A thorough examination of newborn babies at birth establishes the diagnosis of ARM,^3^,^4^ however if the malformation is missed, baby develops gradual abdominal distension which leads to vomiting, bacterial translocation leading to sepsis as well as respiratory distress and aspiration pneumonia.^5^ Missing the anomaly in newborn period results in delayed treatment which can lead to increased morbidity and mortality.^6^

In our practice, in addition to delayed diagnosis of ARM among babies born at home, we receive babies with missed ARM who were born even at tertiary care hospitals and discharged only to be readmitted later. There is no study around the world that determined the frequency of place of delivery and person detecting the anomaly among newborn babies presenting with delayed diagnosis of anorectal malformation. The aim of this study was to determine the place where baby was born and person who detected the anomaly and time taken to identify anomaly so as to plan strategy in providing information to facilitate early recognition of ARM and safe transfer of babies to referral center.

## METHODS

This Descriptive Cross-Sectional Study was conducted at Department of Paediatric Surgery, National Institute of Child Health Karachi, from February 19, 2019 to August 18, 2019. We used the reference of Sinha et al. 2008^7^ who reported that 88.4% (38 out of 43 patients) who presented with delayed ARM presentation had home delivery. Using this value as the anticipated frequency of home delivery among infants with delayed ARM presentation at 6% absolute precision and 95% confidence level, the required sample size turned out to be 110 subjects using WHO sample size calculator. We used Non-probability, Consecutive Sampling technique to collect data. All infants with delayed diagnosis of ARM (diagnosis beyond two hours of life was taken as delayed diagnosis) who visited to outpatient department and emergency of NICH were included in study. Patients with common cloaca & Babies’ parents refusing to participate in study were excluded.

After approval from hospital ethical review committee (IERB No: 07/2018), data collection was started. A written informed consent was taken from parents/guardian of each patient by the primary investigator and all eligible patients were included and enrolled in the study. Complete clinical examination was done for diagnosing anorectal malformations and associated anomalies. All patients were clinically examined for absent anal opening and for presence of associated fistula (Rectoperineal, rectovestibular or rectovaginal in females, Restoperineal or Rectourinary fistula in males). For Patients without associated fistula, Cross Table Lateral Xrays were performed to assess the level of rectal pouch. Ultrasound KUB and Echocardiography was performed to rule out associated genitourinary or cardiac anomalies respectively. All the findings were entered into the pre-designed Proforma. Data was collected on socio-demographic details, mode of delivery (Spontaneous Vaginal Delivery (SVD) / C-Section), place of delivery (home/ hospital) and person detecting the anomaly (medical person/ non-medical person), time of presentation and type of ARM. A questionnaire was filled by primary investigator.

The data was entered and analyze into SPSS Version 20. Mean ± SD and median were calculated for continuous variables. Frequency and percentage were calculated for categorical variables. Diagnostic delay was taken as outcome. Stratification was done on person detecting anomaly with respect to place of delivery to see the effect of these on outcome by using chi-square test considering P ≤ 0.05 as significant.

## RESULTS

In this study 110 patients were included. Out of 110 patients, 76 (69%) were male and 34 (31%) were female. Median age at diagnosis was 48 hours (IQR 24-76). Seven out of 110 patients presented after seven days of life, the details of these patients presenting unusually late are given in Table-I. Seventy-six babies (69.09%) had associated fistulas while 34 patients (30.09%) had no fistula. Among Babies with Fistulae, thirty five babies (46.05%) had rectoperineal fistula, 23 (30.26%) had recto urinary fistula, 15 (19.73%) had recto vestibular while 3 (0.03%) had rectovaginal fistula.

**Table-I:** Patients Diagnosed with ARM beyond 7 days of life.

Sr. No	Age at diagnosis	Gender	Place of Delivery	Person detecting anomaly	Type of anomaly (with ARM)
1	360 hrs ( 15 days)	Female	Teaching hospital	Mother	ACF
2	360 hrs ( 15 days)	Female	Home	Neonatologist	RVF
3	2400 hrs (100 days)	Female	Home	Neonatologist	RVF
4	2880 hrs ( 120 days)	Female	Non teaching hospital	Mother	ACF
5	2880 hrs ( 120 days)	Male	Non teaching hospital	Surgeon	ACF
6	7200 hrs (300 days)	Female	Teaching hospital	Neonatologist (Sec)	ACF
7	43800 hrs (1825 days = 5 years)	Female	Home	Neonatologist	RVF

ACF: Anocutaneous Fistula; Sec: Secondary; RVF: Rectovestibular fistula.

Regarding mode of delivery, Seventy-Seven (70%) patients were delivered through spontaneous vaginal delivery while 33 (30%) were delivered through C-Section. As regards place of delivery, Ninety one (82.7%) patients were delivered at hospital either by C-Section or via SVD while only 19 (17.2%) patients were delivered at home via SVD. Out of 91 hospital deliveries, 11 (12.08%) were born at teaching hospital, 63 (69.23%) at non-teaching hospital while 17 (18.6%) were delivered at maternity care clinics.

**Graph.1:**
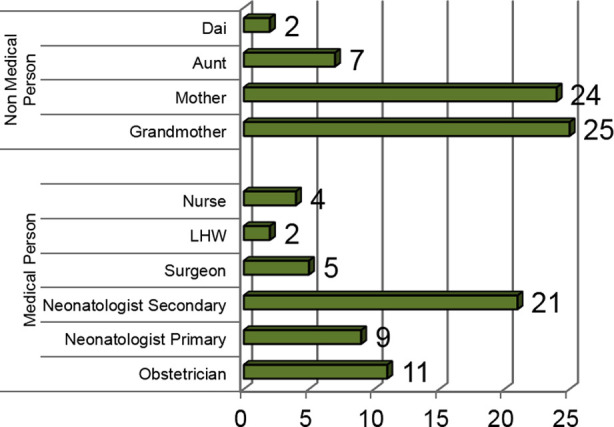
Person Detecting ARM.

Most of the anomalies were detected by non-medical persons n= 58 (52.7%) while medical persons detected anomaly in 52 (47.27%) patients as shown in Graph.1, which shows that number of anomalies detected by non-medical persons were significantly greater than medical persons (P = 0.029). Person who detected the anomaly most of the time was neonatologist n=30 (27.3%) followed by grandmother n=25 (22.7%)**.** Of the 30 patients diagnosed by neonatologists to be having imperforate anus, 9 patients were primarily examined by them at routine birth examination (beyond two hours of birth) while for 21 babies, neonatologists were consulted secondarily by the family, these 21 babies were born at hospital and were discharged without being diagnosed.

**Graph.2:**
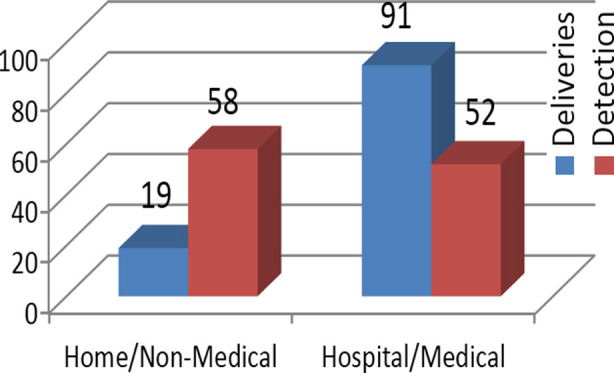
Home delivery & detection (non-med) Vs Hospital delivery & detection(med).

Statistically significant difference was found (P= 0.0001) as shown in Graph-2 where most of deliveries and detection of anomalies were at home by non-medical person against hospital deliveries and detection by medical personal.

## DISCUSSION

Careful perineal inspection during neonatal examination at birth is the key point in identifying ARMs. We used to cut off of two hours in our patients to be diagnosed at neonatal examination that is mandatory for neonatologist, or picked up early within two hours by mother or home member in case of home delivery and consider the older patients as having “delayed diagnosis”.

Our study included 110 patients with delayed diagnosis of ARM which is a huge number in a period of 6 months only. Of these, median age at detection of ARM was 48 hours. Similarly, a study by Beudeker N et al^8^ showed that only 8 (17.39%) of 46 patients with ARM presented during first week after birth while the median age at presentation was 24 days. Contrary to this, Statovci S et al analyzed 76 patients with ARM, of them 18 (23.68%) patients presented beyond neonatal period.^9^

Our data shows that 91 out of 110 (82.8%) patients with ARM were born at hospital and diagnosis was missed while 19 of 110 (17.2%) babies were born at home. Contrary to our results, Sinha SK et al in 2008 reported that 88.4% (38 out of 43 patients) who presented with delayed diagnosis of ARM had home delivery^7^ which points to missed neonatal examination despite more hospital births in our population.

Our results showed tha 58 out of 110 (52.7%) patients were diagnosed by family members at home despite only few babies having home delivery (17.2%) compared to 82.7% deliveries conducted by health care professionals at hospitals and this contradicts previous published literature showing more home births with delayed diagnosis. Of total 110 patients, most of diagnoses were made by grandmothers (22.7%), greatly outnumbering the medical persons as well. These were mostly during changing of clothes and some of them being worried that baby had not passed meconium yet or during massaging of the baby by grandmother which is a cultural heritage in our society.

There are occasional case reports of delayed presentations of these malformations at later age, even in adult life.^10^-^12^ In our series we also had seven of our patients presented beyond seven days of birth. The cause of delayed presentation in these patients was either because of wrong advice regarding the correct age of treatment of these malformations or social factors such as the lack of money, migrated father working out of station, lack of social support and most importantly distance from hospital. The modes of delayed presentation in our setup are different from developed countries, where constipation and abnormally positioned anal opening detected by parents are more common.^13^ One of our patients was a female who presented at age of 5 year with recto vestibular fistula and presented only once she developed straining, constipation and perineal excoriation. Similarly, there are case reports in the literature^14^,^15^ regarding the presentation of ARM in females at adolescence.

Our data shows missed diagnosis by medical persons despite most deliveries (82.7%) being conducted at hospital. Patients were diagnosed by the family which shows significant level of awareness in family members contrary to a study done in Nigeria^16^ which showed low level of awareness of ARM amongst mothers. Bloomfield had reported that even medical specialists might miss an ARM.^17^

From our fairly large experience with delayed diagnosis of ARM, we strongly feel the need of neonatal examination at hospital before discharge. The diagnosis is clinical and can be done by anyone involved in delivery, by quick perineal examination of the baby. Awareness can be provided by showing pictures of absent anal opening. Once diagnosed, baby can be urgently referred to pediatric surgical specialty within first few hours. For this, awareness sessions for non-medical persons and family members in community and workshops & conferences for medical community need to be run so that the anomaly may be diagnosed early to prevent avoidable morbidity and mortality.

### Limitations of the study:

Our study had few limitations as well. One, being a tertiary care referral centre, some of the patients were brought from far off areas which took additional time to diagnose the anomaly however this confounding factor was not assessed in our study.

## CONCLUSION

It is concluded that Non-medical person detected ARM mainly despite the babies being delivered mostly at the hospital, indicating the need for meticulous neonatal examination. However, there is a need to conduct more studies using large sample size with multiple study sites in Pakistan to validate these results. Still there is a room for conducting awareness sessions based on findings of our study to avoid missing this anomaly.

### Authors’ Contribution

**SP** conceived, designed and did statistical analysis & manuscript writing and final reading.

**SA** did data collection, discussion writing and proofreading.

**AJ** did data collection and literature review

**BF** did review and editing of manuscript.
